# The maternal health knowledge of pregnant women attending the antenatal clinics in the Western Cape: A cross-sectional study

**DOI:** 10.4102/jphia.v17i1.1535

**Published:** 2026-05-07

**Authors:** Thabani M. Noncungu, Talitha Crowley, Jennifer Chipps

**Affiliations:** 1 School of Nursing, Faculty of Community and Health Sciences, University of the Western Cape, Bellville, South Africa

**Keywords:** Antenatal care, knowledge, maternal health, pregnant women

## Abstract

**Background:**

Maternal health knowledge enables pregnant women to access, understand and apply health information to support maternal and foetal well-being, while inadequate knowledge may impair caregiving and lead to adverse pregnancy outcomes.

**Aim:**

This study aimed to assess maternal health knowledge and identify associated factors among pregnant women attending antenatal clinics in the Western Cape.

**Setting:**

The study was conducted in three antenatal clinics in the Western Cape, South Africa.

**Methods:**

A descriptive survey was conducted from August 2022 to November 2022 among 248 pregnant women using a researcher-administered questionnaire incorporating the validated 21-item Maternal Health Literacy Inventory in Pregnancy scale. Data were analysed using Statistical Package for Social Sciences (SPSS) version 29. Descriptive statistics and the Mann–Whitney test were employed to examine the associations between demographic characteristics and maternal health knowledge.

**Results:**

Respondents demonstrated low to moderate maternal health knowledge (mean = 58.50/100, standard deviation [s.d.] = 13.79, with over half (51.2%, 95% confidence interval [CI]: 45.0–57.4) classified as having problematic levels of maternal health knowledge. The highest ratings for knowledge were for foetal well-being (mean = 3.33, s.d. = 0.65) and lowest for postnatal care (mean = 3.26, s.d. = 1.17). Higher knowledge score was significantly associated with secondary or higher education (*χ*^2^ = 3.570, *p* < 0.001) and living with others (*χ*^2^ = 4.568, *p* = 0.028).

**Conclusion:**

Maternal health knowledge was inadequate, highlighting the need for strengthened and targeted maternal health education, especially for less-educated women and those living alone.

**Contribution:**

The study provides evidence to guide healthcare practitioners and programme planners in improving maternal health education in antenatal clinics in the Western Cape.

## Introduction

According to United Nations International Children’s Emergency Fund (UNICEF), improving the survival of mothers, new borns and children remains an urgent global challenge, with millions still dying from largely preventable causes despite progress over the last two decades. It highlights persistent inequities and ongoing high maternal mortality, showing that maternal and child health continue to be a major public health concern.^1^ In South Africa, the maternal mortality ratio (MMR) for 2020 was estimated at approximately 109 deaths per 100 000 live births, showing a decline of 33.5% from 2015 (*n* = 164/100 000). However, this is still well above the 2030 target of 70 deaths per 100 000 live births.^2^ In South Africa and other African countries such as Kenya, maternal mortality is because of haemorrhage, eclampsia, sepsis, ruptured uterus, anaemia and heart disease.^3,4^ Maternal health knowledge plays a crucial role in preventing maternal deaths as it enables a woman’s ability to recognise health risks, seek timely care and follow medical guidance during pregnancy, childbirth and the postpartum period. A systematic review has demonstrated that higher levels of maternal health knowledge are directly associated with improved maternal and foetal health outcomes, including reduced risks of low birth weight, preterm birth and obstetric complications.^5^

Maternal health knowledge is defined as a woman’s understanding of health-related topics that affect her during pregnancy, childbirth and the postpartum period.^6^ It includes awareness of healthy practices, risk factors, danger signs and the importance of utilising healthcare services for herself and her baby. Maternal health knowledge is essential for enabling women to understand and monitor their health during pregnancy and can assist pregnant women in understanding the role of healthcare professionals and in appropriate self-care during pregnancy.^7^ However, low levels of maternal health knowledge remain a concern in Africa.^8^ A study conducted in Ethiopia reported that less than half (44.8%) of pregnant women demonstrated knowledge about pregnancy complications, indicating a substantial gap in awareness that could impact access to obstetric care.^9^ Poor maternal health knowledge leads to delayed danger sign recognition, low use of maternal care and higher maternal and neonatal risks.^10^ It may also lead to nutritional problems, poor birth preparedness, the risk of unsafe abortion^11^ and dependence on misinformation or traditional beliefs.^12^ A previous study conducted in Laos revealed that the minority of pregnant women possessed inadequate (*n* = 82, 16.4%) and more than a half were problematic (*n* = 258, 51.6%), with only a third possessing sufficient (*n* = 130, 26%) and excellent (*n* = 30, 6%) knowledge.^13^

Maternal health knowledge is influenced by a range of factors, including gestational age, parity, level of education and the number of previous pregnancies. These variables collectively contribute to variations in pregnant women’s understanding and awareness of maternal health practices. Empirical evidence supports these associations, with previous studies highlighting that education level, parity and gestational age significantly affect maternal health knowledge.^14,15,16^

Even though some studies have been conducted to investigate the level of maternal health knowledge among pregnant women and the relationship to pregnancy outcomes, few studies have been conducted, particularly among pregnant women in South Africa.^17,18^ Investigating maternal health knowledge will help to identify tailored interventions. Therefore, this study aimed to assess maternal health knowledge and identify the factors affecting it among pregnant women attending antenatal clinics in the Western Cape.

## Research methods and design

### Study design

A quantitative cross-sectional study design with both descriptive and analytical components was employed, using a researcher-administered questionnaire to describe maternal health knowledge and examine its association with selected socio-demographic variables.

### Study’s setting

Three antenatal clinics were included in the study, all located in the Northern Tygerberg sub-district, Western Cape. The Northern Tygerberg sub-district has a population of fewer than 424 945 residents, comprising diverse racial and socio-economic groups with an average annual growth rate of 5.31%.^19^ The antenatal clinics are sections or segments of the Midwifery Obstetric Units (MOUs) located on the community health centre (CHC) premises. The antenatal clinics were purposely selected because they offer basic antenatal care and serve a large catchment area of pregnant women from lower socio-economic backgrounds in the Northern Tygerberg sub-district, Western Cape. The antenatal clinics provide an 8-h antenatal care service that includes regular check-ups, health education and promotion, screening tests, monitoring foetal development and supportive services.

### Study population and sampling strategy

The target population was 900 pregnant women attending antenatal clinics during the data collection period. The inclusion criteria for the study were pregnant women (18 years and over) who attended antenatal clinics for antenatal services. The calculator.net sample size calculator was used to calculate the sample size, with the margin of error of 6% and confidence level of *z* = 95% and population proportion of *p* = 50%, suggesting a sample size of 267.^20^ A 6% margin of error was selected to balance statistical precision with feasibility while maintaining acceptable estimate accuracy for this cross-sectional study. Furthermore, the sample size calculated for the descriptive objective was also adequate for the analytical component, as post hoc power analysis demonstrated sufficient power to detect moderate effect sizes at the 5% significance level. Selecting *p* = 50% is statistically conservative and prevents underestimation when the true proportion is unknown. The sample size was calculated as 267 to account for an expected non-response rate, ensuring that the study would still have enough participants for reliable results even if some did not respond. A systematic sampling method was used by selecting every third attendee to be approached for participation in the study. The decision to select every third patient was undertaken to ensure an even distribution of participants while maintaining logistical feasibility for data collection. When the selected third patient declined to participate, a systematic substitution procedure was applied. The study population comprised approximately 900 pregnant women, from which a sample of 267 was required. Using systematic sampling, every third patient was selected. The interval was derived by dividing the total population (*n* = 900) by the sample size (*n* =267), yielding approximately three.

### Data collection tool

The questionnaire included the valid and reliable Maternal Health Literacy Inventory in Pregnancy (MHELIP) scale with 48 statements, of which 21 statements measure maternal health knowledge (21 items).^21^ The maternal health knowledge scale includes statements rated on a 5-point Likert scale from ‘I don’t know at all’ (1), ‘I know a little’ (2), ‘I know something’ (3), ‘I know a lot’ (4) and ‘I know fully’ (5). To calculate the total score, the recommended formula was used (Score = [raw score – minimum possible raw score]/[maximum possible raw score – minimum possible raw score] × 100) and then classified into four categories: inadequate (0 to 50), problematic (50.1 to 66), sufficient (66.1 to 84) and excellent (84.1 to 100). The inadequate and problematic maternal health knowledge categories were defined as limited health literacy (*n* < 66.1/100), while the sufficient and excellent maternal health knowledge categories were defined as desired health literacy (*n* > 66/100). The internal consistency of the maternal health knowledge scale in this sample was calculated for scale reliability and was acceptable (Cronbach’s α = 0.78) although lower than that reported in a previous study by Taheri et al.,^22^ which found a reliability coefficient of α = 0.94. The original questionnaire was adapted into English and subsequently back-translated into isiXhosa to enhance comprehension. The isiXhosa is the dominant language in the study setting; therefore, this language was used for data collection.

### Data collection procedures

Prior to data collection, the questionnaire was pretested at three antenatal clinics within the Northern Tygerberg sub-district. The pretesting of the questionnaire was carried out a month before the main study (July 2022) at the three selected antenatal clinics on 15 pregnant women who met the established inclusion criteria. The pretesting of the questionnaire was conducted to assess the feasibility of the study procedures, refine data collection instruments, evaluate recruitment and logistical processes and identify potential methodological challenges prior to the main study. The respondents on the retesting of the questionnaire were excluded from the main study.

Data were collected at the three antenatal clinics located in the Northern Tygerberg sub-district between August 2022 and November 2022 after obtaining ethics approval from the university and permission from the Department of Health (DOH) and managers of the antenatal clinics. Data were collected in clinic waiting rooms over approximately 30–40 days per clinic, with data collection at each site completed before proceeding to the next clinic. Two trained research assistants with master’s degrees in nursing collected the data between 8:00 and 16:00 each working day during the 4-month period. Research assistants received face-to-face training on the administration of the questionnaire, the provision of study information that included the study’s objectives, potential risks, respondents’ expectations and the procedures for obtaining written informed consent. Standardised data collection procedures, verification of completed questionnaires, data entry checks and strict adherence to predefined inclusion and exclusion criteria were employed to ensure the quality and integrity of data collection. The researcher addressed potential biases by employing a structured questionnaire with a standardised administration protocol, emphasising anonymity and confidentiality to encourage honest responses and utilising systematic sampling to minimise the influence of convenience sampling. The respondents were presented with the study information and provided written informed consent. The researcher and assistants administered the questionnaire to all pregnant women who met the inclusion criteria. Data collection was conducted in the morning during tea time and in the afternoon during lunch time, when the participants were not busy with nurses, to avoid interrupting maternal services. The questionnaire took approximately 25 min to complete.

### Data analysis

The study data were captured and cleaned in the Statistical Package for the Social Sciences (SPSS) version 29.0 for analysis. Age was categorised into two groups: ≤ 35 years and > 36 years. This classification is based on existing literature, which defines advanced maternal age as 35 years and older, while individuals under 35 years are considered to be within the normal reproductive age range. Advanced maternal age has been associated with increased risks during pregnancy and childbirth, including a higher incidence of maternal and neonatal complications such as gestational hypertension and preeclampsia. Categorising participants in this manner facilitates a meaningful analysis of age-related differences in health knowledge, behaviours and outcomes.^23^

The researchers categorised 21 maternal health knowledge statements into four thematic groups to facilitate understanding of the phenomenon: pregnancy and well-being (11 items), foetal development and monitoring (6 items), preparation for labour (2 items) and postnatal care (2 items). Socio-demographic characteristics were presented using frequencies and percentages. In addition, the Likert scale options were recoded as ‘I don’t know’ (0) and ‘I know a lot’ (1). Maternal health knowledge statements were analysed using mean, standard deviation and 95% confidence interval (CI). The Mann–Whitney test was used to test significant associations between maternal health knowledge statements and socio-demographic characteristics to test the hypotheses. The Mann–Whitney (U) test was employed to examine differences between two independent groups because the outcome variable was not normally distributed. The maternal health knowledge was ranked into three categories, namely inadequate (0–50/100), problematic (50.1 to 66/100), sufficient (66.1–84/100) and excellent (85 and above). Combining sufficient and excellent simplifies the presentation of results without losing meaningful distinctions relevant to the research objectives. The ‘sufficient’ and ‘excellent’ categories were combined into the ‘sufficient to excellent’ (66.1 – 100) category (called desired level of maternal health knowledge) because of the small number (*n* = 6, 2.4%) of respondents in the ‘excellent’ category. There were no missing data in the population. Therefore, there was no need for the researchers to address the bias.

### Ethical considerations

Ethics approval was obtained from the University of the Western Cape Humanities and Social Sciences Research Ethics Committee (HSSREC), reference number REC: BM21/02/05. Permission to conduct the study was sought from the Western Cape Department of Health (DOH), sub-district manager and antenatal clinic managers. Participation in the survey was voluntary, and no personal information was collected from respondents. Participants were informed about their voluntary participation, that their responses were anonymous and that they could easily withdraw from the survey at any given time.

## Results

### Socio-demographic characteristics

The results show the socio-demographic characteristics according to parity of 248 pregnant women (response rate: *n* = 248/267, 92.8%) who provided informed consent to participate in this study (Table 1). The mean age of the respondents was 27.4 years (standard deviation [s.d.] = 7.53), with most of the respondents (*n* = 204, 82.3%) being less than 35 years old. More than half (*n* = 133, 53.6%) of the respondents did not have a partner, and the majority of the respondents (*n* = 222, 89.5%) had attained secondary education and higher. More than half (*n* = 126, 50.8%) of the respondents did not know the gestational age of their pregnancy. Over half (*n* = 127, 52.2%) of the respondents were employed, and three-quarters (*n* = 186, 75.0%) of the respondents lived with other people, such as family and the baby’s father (Table 1). Significant differences were observed between maternal health knowledge and level of education (Table 1). Respondents who had secondary education and higher were more likely to have low maternal health knowledge than their counterparts who had primary education (89.4 vs. 10.6, *χ*^2^ = 3.570, *p* < 0.001). Furthermore, a significant association between maternal health knowledge and living status, where the respondents who live alone were more likely to have low maternal health knowledge than their counterparts who live with others (75.8 vs. 24.2, *χ*^2^ = 4.568, *p* < 0.028).

**TABLE 1:** Socio-demographic characteristics and maternal health knowledge (*N* = 248).

Variables	Desired maternal health knowledge				All number (*n* = 248, 100%)		Chi-square (*χ*^2^)	*p*-value
	No (*n* = 182)		Yes (*n* = 66)					
	*n*	*%*	*n*	*%*	*n*	*%*		
**Age in years**	**-**	**-**	**-**	**-**	**-**	**-**	1.071	0.790
< 35	149	81.9	55	83.3	204	82.3	-	-
> 36	33	18.1	11	16.7	44	17.7	-	-
**Marital status**	**-**	**-**	**-**	**-**	**-**	**-**	1.464	0.226
No partner	143	78.6	47	71.2	190	76.6	-	-
Partner	39	21.4	19	28.8	58	23.4	-	-
**Level of education**	**-**	**-**	**-**	**-**	**-**	**-**	3.570	0.001*
Primary	19	10.4	7	10.6	26	10.5	-	-
Secondary and higher	163	89.6	59	89.4	222	89.5	-	-
**Knowledge of gestational age**	**-**	**-**	**-**	**-**	**-**	**-**	1.030	0.467
Don’t know	95	52.2	31	47.0	126	50.8	-	-
Knowledgeable	87	47.8	35	53.0	122	49.2	-	-
**Employment status**	**-**	**-**	**-**	**-**	**-**	**-**	3.178	0.075
Employed	95	52.2	46	69.7	127	51.2	-	-
Unemployed	87	47.8	20	30.3	121	48.8	-	-
**Living status**	**-**	**-**	**-**	**-**	**-**	**-**	4.568	0.028*
Living alone	46	25.3	16	24.2	62	25.0	-	-
Living with others	136	74.7	50	75.8	186	75.0	-	-

* , Denotes a significant *p*-value of *p* < 0.05

### Maternal health knowledge

The maternal health knowledge subscale was measured using 21 statements, with higher mean scores indicating higher levels of self-rated maternal health knowledge. The overall score for maternal health knowledge was: 58.50 out of 100, s.d. = 13.79, range: 13–100. More than half of the respondents (*n* = 127; 51.2%, 95% CI: 45.0–57.4) were categorised as possessing problematic maternal health knowledge, with scores ranging from 50.1 to 66 (Table 2).

**TABLE 2:** Maternal health knowledge categories (*N* = 248).

Categories	1st pregnancy (*n* = 98, 39.5%)		2nd pregnancy and more (*n* = 150, 60.5%)		Total	
	*n*	*%*	*n*	*%*	*n*	*%*
Inadequate	23	23.5	32	21.3	55	22.2
Problematic	53	54.1	74	49.3	127	51.2
Sufficient to excellent	22	22.4	44	29.3	66	26.6

**FIGURE 1:**
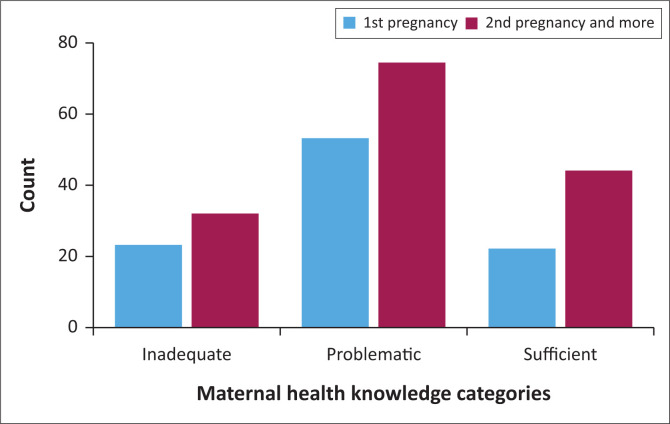
Knowledge scores of maternal health by number of pregnancies (*N* = 248).

An additional 55 participants (22.2%, 95% CI: 17.0–27.4) demonstrated inadequate knowledge (scores between 0 and 50), while 66 respondents (26.6%, 95% CI: 21.1–32.1) exhibited sufficient to excellent knowledge, scoring between 66.1 and 100 (see Table 2). A higher proportion of the respondents with problematic maternal health knowledge were in their second or subsequent pregnancy (*n* = 74, 58.2%) compared to their first pregnancy (*n* = 53, 41.7%) (Figure 1), but the differences were not statistically significant (Table 3). Similarly, no significant differences were found between maternal health knowledge and level of education and gestational age.

**TABLE 3:** Maternal health knowledge statements.

Statements	1st pregnancy		2nd pregnancy or more		Mean	s.d.	95% CI	Mann–Whitney (*U*)	*p*-value
	Mean/4	s.d.	Mean/4	s.d.					
**Pregnancy and well-being**	-	-	-	-	3.33	0.65	3.25–3.49	-	-
Natural physical changes during pregnancy	3.76	1.23	3.74	1.11	3.75	1.15	3.60–3.89	71.17	0.657
Natural psychological changes during pregnancy	3.50	1.31	3.66	1.17	3.60	1.23	3.44–3.75	78.27	0.372
Proper nutrition during pregnancy	3.34	1.23	3.44	1.28	3.40	1.26	3.24–3.56	77.66	0.439
Acceptable and normal amount of weight gain during pregnancy	3.07	1.23	3.54	1.16	3.35	1.20	3.20–3.51	89.46	0.003*
Personal healthcare	3.29	1.32	3.33	1.20	3.31	1.24	3.15–3.47	74.50	0.868
Common pregnancy problems such as nausea, vomiting and lower backpain	2.89	1.23	3.51	1.19	3.27	1.24	3.11–3.42	94.22	0.001*
Pregnancy supplements (vitamins)	3.09	1.45	3.30	1.37	3.22	1.40	3.04–3.39	79.42	0.273
Proper sexual relation during pregnancy	3.18	1.25	3.22	1.30	3.21	1.28	3.05–3.37	74.58	0.840
Injecting safe (allowed) vaccines during pregnancy	3.00	1.13	3.35	1.17	3.21	1.16	3.07–3.36	86.10	0.019*
Proper activity and status in pregnancy	3.11	1.32	3.17	1.31	3.15	1.33	2.98–3.32	75.29	0.740
Proper exercise during pregnancy	3.00	1.30	3.25	1.35	3.15	1.33	2.88–3.30	81.40	0.143
**Foetal development and monitoring**	-	-	-	-	3.40	0.77	3.30–3.49	-	-
Appropriate referral timing for pregnancy examinations (visits)	3.60	1.16	3.75	1.21	3.69	1.18	3.54–3.84	80.14	0.210
Diagnostic examination (ultrasound and tests) of maternal and foetal health in pregnancy	3.47	1.12	3.79	1.07	3.67	1.10	3.53–3.80	85.14	0.025*
Pregnancy disease symptoms such as gestational diabetes, high blood pressure in pregnancy and other diseases	3.48	1.42	3.28	1.35	3.36	1.37	3.19–3.53	66.60	0.200
Risk signs in pregnancy	3.53	1.30	3.12	1.35	3.28	1.34	3.11–3.45	60.59	0.017*
Factors affecting foetal health such as photography, medications, chemicals such as botox, etc.	3.26	1.25	3.22	1.30	3.23	1.28	3.07–3.39	72.44	0.845
Normal number of foetal movements	3.35	1.27	3.01	1.36	3.14	1.34	2.97–3.31	63.05	0.053
**Preparation for labour**	-		-	-	3.32	1.06	3.19–3.46	-	-
Methods of pain relief in vaginal delivery	3.20	1.34	3.41	1.23	3.33	1.27	3.17–3.49	80.05	0.223
Childbirth such as the advantages and disadvantages of each of the natural delivery methods and caesarean section and their associated care	3.36	1.20	3.30	1.31	3.32	1.26	3.16–3.48	72.47	0.848
**Postnatal care**	-	-	-	-	3.26	1.17	3.11–3.40	-	-
Required postpartum care of mother	3.34	1.37	3.27	1.25	3.30	1.29	3.14–3.46	70.84	0.621
Neonatal and infant care in the postpartum period	3.14	1.38	3.26	1.30	3.21	1.33	3.05–3.38	76.97	0.520

CI, confidence interval; s.d., standard deviation.

* , Denotes a significant *p*-value of *p* < 0.05.

### Maternal health knowledge domains

**FIGURE 2:**
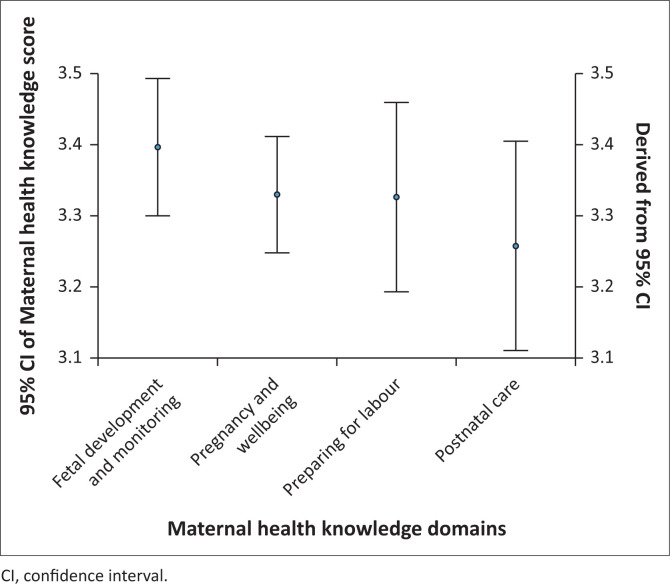
Comparison of maternal health knowledge domains (95% confidence intervals).

Respondents rated their knowledge on *foetal development and monitoring* domain the highest 3.4 (s.d. = 0.77), (95% CI: 3.30–3.49); followed by *pregnancy and well-being* 3.33 (s.d. = 0.65), (95% CI: 3.25–3.41); and *preparing for labour* 3.22 (s.d. = 1.06), (95% CI: 3.19–3.46). The lowest rated is the *postnatal care* 3.26 (s.d. = 1.17), (95% CI: 3.11–3.40). There were no significant differences between the average knowledge scores for the domains of knowledge (Figure 2). The comparison is limited to these domains, and the overlap of the 95% CIs indicates the relative similarity in maternal health knowledge levels across domains, rather than statistically tested differences between them.

### Pregnancy health and well-being

*Pregnancy health and well-being* knowledge was rated with 11 statements, with an overall mean of 3.33, (s.d. = 0.65), (95% CI: 3.25–3.41). The highest rated maternal health knowledge statement was ‘I know natural physical changes during pregnancy’ 3.75 (s.d. = 1.15), (95% CI: 3.60–3.89). This was followed by the statement ‘I know natural psychological changes during pregnancy’ 3.60 (s.d. = 1.23), (95% CI: 3.44–3.75). The lowest-rated statements were ‘I know proper activity and status in pregnancy’ (*M* = 3.15, s.d. = 1.33), (95% CI: 2.98–3.32) and ‘proper exercise during pregnancy’ (*M* = 3.15, s.d. = 1.33), (95% CI: 2.88–3.30).

Respondents with more than one pregnancy (*M* = 3.54, s.d. = 1.16) rated their knowledge of the acceptable and normal amount of weight gain during pregnancy significantly higher than first-time pregnant respondents (*M* = 3.07, s.d. = 1.23), *U* = 89.46, *p* = 0.003). Similarly, they rated knowledge of common pregnancy-related problems such as nausea, vomiting and lower back pain, significantly higher (*M* = 3.31, s.d. = 1.19) than those in their first pregnancy (*M* = 2.89, s.d. = 1.23) (*U* = 94.22, *p* = 0.001) and knowledge of the safety and appropriateness of vaccines during pregnancy (*M* = 3.35, s.d. = 1.17 vs. *M* = 3.00, s.d. = 1.13), respectively (*U* = 86.10, *p* = 0.019) (Table 3). There was a relationship between the pregnancy health and well-being items and number of pregnancies.

### Foetal development and monitoring

*Foetal development and monitoring* were rated the most knowledgeable area (*M* = 3.40, s.d. = 0.77), (95% CI: 3.30–3.49). The highest rated statement was ‘I know the appropriate referral timing for pregnancy examinations (visits)’ (*M* = 3.69, s.d. = 1.18), (95% CI: 3.54–3.84), and the lowest-rated statement was ‘I know the normal number of foetal movements’ (*M* = 3.14, s.d. = 1.34), (95% CI: 2.97–3.31) (Table 3). Respondents with more than one pregnancy rated knowledge of diagnostic examinations (e.g. ultrasound and laboratory tests) significantly higher than those with a first pregnancy (*M* = 3.79, s.d. = 1.07 vs. *M* = 3.47, s.d. = 1.12), respectively (*U* = 85.14, *p* = 0.025). However, respondents with a first pregnancy rated their knowledge of warning or risk signs during pregnancy higher than those with more than one pregnancy (*M* = 3.53, s.d. = 1.30 vs. *M* = 3.12, s.d. = 1.35), respectively (*U* = 60.59, *p* = 0.017) (Table 3). There were no significant differences were found for level of education and gestational age.

## Preparation for labour, postnatal care and neonatal and infant care

*Preparation for labour* (*M* = 3.32, s.d. = 1.06), (95% CI: 3.19–3.46) and *postnatal care* (*M* = 3.26, s.d. = 1.17), (95% CI: 3.11–3.40) were rated the lowest knowledge areas. The highest rated statement for preparation for labour was ‘I know the methods of pain relief in vaginal delivery’ (*M* = 3.33, s.d. = 1.27), and ‘I know required postpartum care of mother’ (*M* = 3.30, s.d. = 1.29), (95% CI: 3.14–3.46) for postnatal care (Table 3). The lowest-rated maternal health knowledge item pertained to neonatal and infant care during the postpartum period (*M* = 3.14, s.d. = 1.38), (95% CI: 3.05–3.38). No significant associations were found between preparation for labour, postnatal care and socio-demographic characteristics.

## Discussion

### Overall maternal health knowledge

This study provides valuable insights into the existing body of knowledge on maternal health literacy. The primary outcome of this study was maternal health knowledge, measured using 21 items of the MHELIP scale. There are few studies conducted in Africa that specifically used maternal health knowledge of MHELIP scale. One study that employed the MHELIP scale conducted among pregnant women in Eswatini found that higher levels of maternal health literacy were significantly associated with more appropriate utilisation of antenatal care services, compared to women with lower health literacy.^24^ A second study that used the MHELIP scale, conducted in Nigeria, discovered that about 41.6% of respondents had inadequate maternal health literacy.^25^ The two studies demonstrated socio-demographic characteristics comparable to those of this study, particularly with respect to the pregnant women’s levels of education, employment status and age.

In this study, respondents exhibited a problematic level of maternal health knowledge, with an overall mean score of 58.50 out of 100 (s.d. = 13.79; range: 13–100), indicating inadequate to problematic health knowledge among 182 participants (73.4%). This finding contrasts with a study conducted in Turkey, which reported that pregnant women possessed a sufficient level of maternal health knowledge, with an average score of 68.44.^26^ This is also supported by the finding that over a half (50.8%) of the respondents did not know the gestational age of the current pregnancy. Not knowing gestational age is inconsistent with studies conducted in Ethiopia and United States, which reported that most pregnant women were aware of the gestational age of pregnancy and were accurate in estimating their pregnancy duration.^9,27^ This finding suggests that variations in overall maternal health literacy and that knowledge of gestational age may be an indicator of overall maternal health literacy in some settings.

### Pregnancy and well-being

Most pregnant women in our study rated their knowledge regarding natural physical changes during pregnancy as the highest 3.75 (s.d. = 1.15). A study conducted in Saudi Arabia concurs with this study, approximately 82% of the women recognised mood swings as a normal symptom of pregnancy, 80.1% were aware that nausea commonly occurs, 75.9% identified fatigue as a typical symptom and 68.9% knew that fainting or dizziness can also be experienced during pregnancy.^28^ This suggests that most pregnant women in this study had a reasonably good level of knowledge about the natural physical changes that occur during pregnancy. The result not only reflects a positive trend in maternal health knowledge but also suggests that education on pregnancy-related body changes should continue to ensure all pregnant women are well informed, especially those who may fall below the average knowledge level.

Our findings revealed that pregnant women rated knowledge of physical activity and exercise in pregnancy as the lowest 3.15 (s.d. = 1.33). In agreement with this study is a study conducted in Iraq that reported nearly two-thirds (60%) of women had poor knowledge levels about physical exercise during pregnancy.^29^ This suggests that pregnant women in this study have a poor understanding of the normal physiological adaptations of pregnancy and physical activity, although some variation remains, indicating the potential benefit of reinforcing educational support during antenatal care. The lack of knowledge on proper activity during pregnancy might lead to risks of problems such as excessive weight gain, pregnancy conditions (gestational diabetes and hypertension disorders) and decreased stamina for labour and delivery. This low knowledge in both studies may be attributed to limited antenatal health education, inadequate counselling by healthcare providers and cultural beliefs that discourage physical activity during pregnancy.

### Foetal development and monitoring

Knowledge regarding the appropriate timing for referral during pregnancy examinations was rated relatively high, 3.69 (s.d. = 1.18). In contrast with the study conducted in Ethiopia, 48% of the respondents reported that they did not know the right time and purpose of making the antenatal booking.^30^ Knowledge of appropriate referral timing for pregnancy examinations is instrumental to prevent complications for maternal and neonatal death. We found that pregnant women rated knowledge on the normal number of foetal movements as the lowest 3.14 (s.d. = 1.34). Similarly, another study conducted in Indonesia reported that more than three-quarters (*n* = 136, 79.4%) of the respondents had limited or poor knowledge about the normal number of foetal movements.^31^ The finding of this study is of concern as limited/diminished foetal movement is a danger sign of pregnancy and missing it might result in foetal death. The difference could be because of the differences in the timing of antenatal check-up. There is a need to empower pregnant women about the normal number of foetal movements and accelerate efforts to prevent complications of pregnancy that lead to death. The empowerment will assist in achieving the Sustainable Development Goal number 3, which seeks to ensure health and well-being for all.

### Preparation for labour

The study’s results indicated that pregnant women rated their knowledge of pain relief methods during vaginal delivery as the highest 3.33 (s.d. = 1.27). Similarly, a South African study found that more than half (*n* = 200, 52.1%) of pregnant women lack knowledge regarding non-pharmacological pain relief methods.^32^ The finding in this study indicates that pregnant women might be able to request pain relief methods to help them cope during labour, which could make the delivery process easy and without complications.

### Postnatal care

Knowledge of postpartum care of the mother was rated among the lowest 3.30 (s.d. = 1.29). The findings of this study are inconsistent with those of a study conducted in Ethiopia, which revealed that postpartum mothers were knowledgeable about postpartum topics such as heavy vaginal bleeding and healthy, balanced foods.^33^ The current results show that the most of pregnant women demonstrated lower understanding of the knowledge required for postpartum care of the mother. This suggests that while many women are aware of essential aspects of pregnancy and well-being, there remains a need for strengthened education and support to ensure comprehensive preparedness for the postpartum period.

Another contrasting finding comes from a study conducted in Nigeria among pregnant women attending specialist hospitals, which reported that participants demonstrated a significant level of knowledge regarding postnatal care.^34^ Although the Nigerian study was conducted among pregnant women attending specialist hospitals, its reported level of postpartum care knowledge is comparable to that observed in this study, which indicates that pregnant women remain largely unaware of key aspects of postpartum care, including physical recovery, mental health and the importance of follow-up medical visits.

### Neonatal and infant care

Lastly, the lowest-scoring item on the maternal health literacy scale in the postpartum period pertained to neonatal and infant care (3.14, s.d. = 1.38). A study conducted in Ghana revealed that more than a half (50.7%) of the mothers had good knowledge about the essential new-born care.^35^

## Conclusion

This study provided valuable insights into the existing literature on maternal health knowledge. Given the scarcity of information on this topic in South Africa, our data serve as a baseline for future comparative studies on the matter. The study found that most pregnant women had inadequate to problematic level of maternal health knowledge. Although the respondents demonstrated a high mean score in the foetal development and monitoring domain, as indicated by the error bar (Figure 2), their knowledge of normal foetal movement within this domain was limited. This indicates that there are knowledge gaps among pregnant women in relation to preparation for labour and postnatal care.

### Recommendations

The midwives/nurses working at antenatal clinics in the Western Cape should provide pregnant women with information on different aspects of maternal health knowledge, such as pregnancy and wellness, foetal development and monitoring, preparation for labour and postnatal care. Future studies should endeavour to include pregnant women attending antenatal clinics in other sub-districts. The antenatal units should be teaching pregnant women on the identification of normal foetal movements during pregnancy, as well as on factors influencing foetal health, such as medication use and pregnancy-related disease symptoms. According to the National Integrated Maternal and Perinatal Care Guidelines, these symptoms are recognised as danger signs of pregnancy, indicating potential complications that require timely intervention.^36^ Further research is warranted to explore the perspectives of pregnant women, and maternal health knowledge should be systematically incorporated into nursing practice.

### Strengths of the study

This is one of the few studies conducted in South Africa to assess maternal health knowledge of pregnant women using the MHELIP scale, with 48 statements, of which 21 statements measure maternal health knowledge (21 items). A key strength of this study lies in its focus on maternal health knowledge among pregnant women, an area in which limited research has been conducted in the region, particularly using this measurement scale.

### Limitations of the study

The study was limited to pregnant women attending three antenatal clinics in the Northern Tygerberg Sub-district, which may not represent the broader population of pregnant women in other regions or healthcare settings leading to selection bias. Furthermore, pregnant women who did not attend antenatal care were excluded from the study. Future studies should endeavour to include pregnant women attending antenatal clinics in other sub-districts. There could have been potential bias observed in the results. The respondents were recruited from clinic attendees, potentially excluding women who do not access antenatal care services or those receiving care from non-facility providers. This may introduce selection bias and over-represent women who are more health-conscious or have better access to care.
